# Evolution of Gas Film and Corresponding Drag Reduction Performance in Microchannels with Multi-Configuration Wall Microstructures

**DOI:** 10.3390/ma19112282

**Published:** 2026-05-28

**Authors:** Hongfei Wang, Ruiyang Li, Zhenya Liu, Dayong Li

**Affiliations:** School of Electromechanical and Automotive Engineering, Yantai University, Yantai 264005, Chinaliuzhenya00@163.com (Z.L.)

**Keywords:** gas film, drag reduction, slip length, microstructures, microchannel

## Abstract

The presence of a gas film at the solid–liquid interface can effectively reduce fluid flow resistance. This study utilizes numerical simulations to explore how groove microstructures on the lower wall of microchannels affect the evolution of trapped bubbles into gas films, as well as the resultant fluid flow behaviors inside microchannels. The influences of groove number, groove shape and double-layer microstructures on gas film formation and fluid boundary slip length are analyzed in detail. The results show that increasing the groove number can significantly improve the continuity and stability of the gas film. Groove shape also has a prominent effect on the evolution of bubbles and gas films. With the longest spreading length of gas film, rectangular grooves present a slip length about 23.9% higher than that of triangular grooves. Simultaneously, it was discovered that bilayer microstructures, especially in cases with smaller periods, can notably enhance the spreading speed, length and thickness of gas film. This improvement, in turn, further increases the slip length and boosts the drag reduction effect. This work highlights that rational optimization of wall microstructures in superhydrophobic microchannels facilitates the generation of stable, continuous gas films, yields a prominent enhancement in slip length, and consequently achieves efficient drag reduction in microfluidic systems. The present findings offer valuable fundamental understanding and technical guidance for the structural design of advanced microfluidic devices.

## 1. Introduction

The resistance of fluids at the solid–liquid interface plays a crucial role in the precise control of fluid dynamics at the micro- and nanoscale. Therefore, developing effective strategies to reduce this resistance remains a critical challenge [1,2,3]. Studies have demonstrated the existence of fluid boundary slip at the solid–liquid interface [4,5,6], which effectively reduces frictional resistance when fluids flow over solid surfaces. However, this behavior is not universal. Some researchers have observed the no-slip boundary condition on smooth hydrophilic surfaces [7], where strong adhesive interactions between the fluid and solid surface prevent slip. In contrast, hydrophobic surfaces, which can adsorb gas molecules, form bubbles that induce slip when liquids flow over the gas-covered surface [8,9,10,11,12]. This discrepancy highlights the critical role of surface wettability and gas adsorption in determining the flow behavior. Bubbles at the solid–liquid interface can effectively convert solid–liquid contact into gas–liquid contact, thereby reducing fluid flow resistance [13,14,15,16,17,18].

Factors such as surface roughness and wall wettability have a significant influence on bubble nucleation and stability [19,20,21]. As a result, extensive studies have been conducted to explore the performance of surfaces with microstructures designed to control bubble distribution, with the goal of optimizing drag reduction [22,23,24,25,26,27,28,29]. On hydrophobic or superhydrophobic surfaces with microstructures, bubbles can stably adhere and even form a continuous gas film, which plays a critical role in drag reduction. Numerical and experimental investigations by Wang et al. [30] revealed that complete gas coverage on grooved surfaces suppresses turbulent kinetic energy and improves drag reduction. Within the flow velocity range of 10–20 m/s, the drag reduction performance of superhydrophobic grooved surfaces increases with rising flow velocity. Moreover, the dynamic evolution of the interfacial gas structure under turbulent flow induces time-varying drag characteristics. Building on the multiphase-flow theoretical framework, Tanaka et al. [31] analyzed the physical mechanisms of bubble growth. Their study highlighted the combined effects of buoyancy forces, slip behavior, and wall wettability conditions on the evolution of the gas–liquid interface. Zhang et al. [32] further explored the factors affecting gas film formation under laminar and turbulent flow regimes. Through comparative investigations of water flow over hydrophilic and hydrophobic surfaces with and without step structures, they confirmed that stable gas films can only form on stepped hydrophobic surfaces. Moreover, optimization of the step height and spacing enabled a maximum drag reduction rate of 20%. Du et al. [33] proposed a dynamic gas film maintenance technology based on air injection. Through flow field analysis, they demonstrated that the uneven roughness of microstructures in the Wenzel state (a fully wetted regime where liquid completely penetrates into microscale groove cavities, resulting in full solid–liquid contact without entrapped gas; this state usually causes increased flow resistance and weakened drag reduction performance) on superhydrophobic surfaces leads to additional energy dissipation, increasing the frictional drag coefficient. In contrast, air injection induces recovery of the Cassie state, re-establishing effective slip boundary conditions on the surface and achieving up to a 20% improvement in drag reduction efficiency by suppressing the generation of turbulent vortices. Yang et al. [34] constructed micro–nano composite surfaces with varying roughness levels and investigated their effects on gas film stability and regeneration. Their experimental results demonstrated that composite-structured surfaces significantly enhance the stability of gas films. Inspired by biomimetic structures, Yao et al. [35] employed femtosecond-laser technology to fabricate alternating superhydrophobic/hydrophobic surfaces, further enhancing the stability of gas films.

The use of trapped bubbles at microstructures for drag reduction on hydrophobic microchannel surfaces offers promising potential for practical applications. The key to optimizing drag reduction lies in the formation of stable, continuous gas films through these trapped bubbles. However, the law of quantitative influence of microstructure geometry on gas film dynamic evolution and boundary slip characteristics still lacks systematic elucidation, which limits the optimal design of high-performance superhydrophobic microchannels. To fill this research gap, this study aims to systematically investigate the effects of different microstructure geometries on gas film formation and stability, and to establish the quantitative relationship between gas film spreading behavior and slip length. This study employs a two-phase-flow model and phase-field method to investigate the factors influencing gas film formation on microchannels with varying microstructure geometries. Additionally, we explore the relationship between gas film spreading and the slip length, providing insights into how wall microstructures can enhance the stability of gas films. This research provides a new perspective on reducing fluid flow resistance at the solid–liquid interface, with significant implications for the design of more efficient microfluidic system applications.

## 2. Simulation Methodology

### 2.1. Model and Simulation Settings

This study focuses on the laminar flow characteristics, gas film evolution mechanism, and drag reduction performance of liquid flow in superhydrophobic grooved microchannels under specific flow ranges. The flow regime investigated in this work is limited to a typical microscale laminar state with Reynolds numbers covering 23.8–238 (the core flow conditions and geometric specifications of the microchannel simulation are summarized in Table 1 for clarity). To systematically explore the influences of groove quantity, groove shape, and dual-layer microstructures on bubble trapping, gas film formation, and internal fluid flow behavior of microchannels, numerical simulations are performed using COMSOL 5.6.

**Figure 1:**
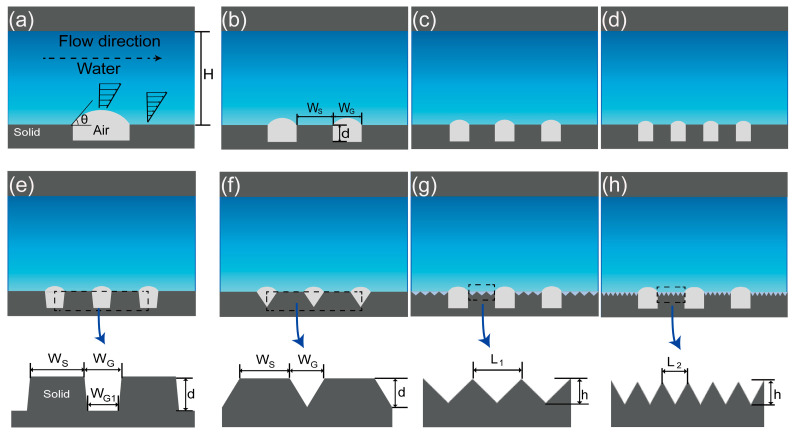
Diagram of the microchannel configuration. (**a**) single groove; (**b**) two grooves; (**c**) three grooves; (**d**) four grooves; (**e**) sosceles trapezoidal grooves; (**f**) isosceles triangular grooves; (**g**) and (**h**) show the double-layer rectangular groove structures, where the periodic lengths of the second-layer microstructures are *L*_1_ = 1 μm and *L*_2_ = 0.5 μm, respectively. The lower wall is equipped with rectangular microstructures. The trapped bubbles in the grooves exhibit an initial protrusion angle of 60°. A no-slip boundary condition is applied at the solid–liquid interface, while a slip boundary condition is specified for the gas–liquid interface.

As shown in Figure 1, microchannel models with groove microstructures of varying shapes and quantities on the lower wall were designed. Figure 1a–d depict microchannels with different groove configurations: a single groove (6 μm in width), two grooves (each 3 μm in width and spaced 4 μm apart), three grooves (each 2 μm in width with a 3 μm spacing), and four grooves (each 1.5 μm in width and spaced 2 μm apart), respectively. The total length (L) of each microchannel model is 20 μm, with a height (H) of 12 μm. The lower walls of the models in Figure 1e,f feature periodically arranged isosceles trapezoidal and isosceles triangular grooves, respectively. Rectangular, trapezoidal, and triangular grooves are selected in this study because imperfect microfabrication of rectangular grooved microchannels commonly produces approximate trapezoidal or triangular cavity profiles in practical engineering. These three typical configurations can well represent the actual morphological features of grooved microstructures fabricated in microfluidic devices. For the isosceles triangular grooves, both the width (WG) and central depth (d) are 2 μm. The bottom base width (WG1) of the isosceles trapezoidal grooves is 1.8 μm, and the periodic spacing (WS) for both structures is 3 μm. Figure 1g,h illustrate double-layer rectangular groove structures, where triangular microstructures are decorated on the ridges of primary rectangular grooves with periodic lengths of *L*_1_ = 1 μm and *L*_2_ = 0.5 μm, respectively. The superimposed secondary microstructures reconstruct the original smooth-ridge morphology of primary grooves, increase the overall surface roughness, and form abundant multiscale microcavities. Such a structural design effectively enriches the surface morphological characteristics and provides more gas-trapping sites to optimize gas film evolution. The height (*h*) of all decorated triangular microstructures is 0.25 μm.

In general, bubbles inside microstructures can be formed through two typical ways: residual air trapping on rough hydrophobic surfaces during liquid filling, and active gas injection. The present study mainly focuses on the effects of microstructure morphology and Reynolds number on bubble spreading, gas film evolution and boundary slip characteristics. Therefore, in the numerical model, initial gas bubbles with a contact angle of 60° are pre-set inside the groove cavities.

It should be noted that this numerical model neglects gas solubility and gas–liquid mass transfer. For short-term microchannel laminar flow, gas dissolution is negligible and exerts little impact on bubble and gas film evolution. This simplification eliminates mass transfer interference, allowing the inherent relationships between microstructure parameters, flow conditions and gas film drag reduction performance to be clearly identified. In practical long-term operation, however, persistent gas dissolution gradually consumes cavity-trapped gas, impairs gas film coverage and stability, and further weakens boundary slip and drag reduction performance.

### 2.2. Boundary Conditions

The upper and lower wall surfaces of the microchannel (with no bubbles) are set as no-slip boundary conditions [25], as described in Equation (1). It is assumed that slip occurs at the gas–liquid interface of the bubbles, with the gas–liquid interface defined as a slip boundary condition, as expressed by Equation (2).

(1) u=0, 

u⋅n=0,

(2) $$k-\left(k\;\cdot \;n\right)n=0,\;$$

Here, n represents the normal vector, and the viscous stress vector $k=\left[\mu \left(\nabla u+{\left(\nabla u\right)}^{T}\right)\right]n$. Under these boundary conditions, the frictional force exerted by the gas layer on the liquid flow is zero, but it restricts the fluid velocity parallel to the wall surface. The inlet boundary condition is set to fully developed flow, which can be expressed as

(3) $$\begin{array}{c} \left[-PI+\mu (\nabla u+{\left(\nabla u\right)}^{T})\right]n=-P_{grad}\cdot n\\ u\cdot t=0 \end{array}$$

At the outlet, the fluid exhibits traditional flow with no backflow, and the boundary condition can be written as [25]

(4) $$\begin{array}{c} n^{T}\left[-PI+\mu (\nabla u+{\left(\nabla u\right)}^{T})\right]n=-{\hat{P}}_{0}\\ u\cdot t=0 \end{array}$$

where $\hat{P}$_0_ is the pressure on the external side of the fluid near the outlet, with $\hat{P}$_0_ ≥ *P*_in_ at the inlet, and the pressure of the fluid at the outlet, with $\hat{P}$_0_ ≤ *P*_out_ at the outlet; **t** is the tangential vector of the wall.

### 2.3. Governing Equations

The fluid flow is governed by the Navier–Stokes equations, and their detailed formulations are given as follows [25]:

(5) $$\rho (u\cdot \nabla )u=\nabla \cdot \left[-PI+\mu (\nabla u+{\left(\nabla u\right)}^{T})\right]+F$$

(6) ρ∇⋅u=0

Here, *p* represents the driving pressure and *F* denotes the external force. The simulation is conducted under constant conditions at 293.15 K, with the water’s density ρ = 0.998 × 10^3^ kg/m^3^, and the dynamic viscosity of the fluid *μ* = 1.005 × 10^−3^ Pa∙s.

Microfluidic flows are dominated by strong interfacial effects. To describe the dynamic evolution of the gas–liquid interface, the phase-field method is adopted, which is governed by the classic phase-field governing equations [20]:

(7) $$\frac{\partial \phi }{\partial t}+u\cdot \nabla \phi \;=\nabla \cdot \left(\epsilon_{pf}^{2}\nabla \psi \right)$$

The phase field variable (ϕ) represents the position of the gas–liquid interface, with values typically ranging from −1 (indicating the gas phase) to 1 (indicating the liquid phase). u is the velocity field, ϵ_pf_ is a parameter related to the interface thickness, and the chemical potential (ψ) represents the gradient of the system’s free energy density and governs the shape and evolution of the interface. Following the free-energy minimization principle, it homogenizes interfacial energy differences, suppresses interfacial fluctuations, and drives the interface to a stable state with minimized total system free energy. Its expression is given by [20]

(8) $$\psi =-\nabla \cdot \left(\epsilon_{pf}^{2}\nabla \phi \right)+\left(\phi^{2}-1\right)\phi +\frac{\epsilon_{pf}^{2}}{\lambda }\frac{\partial f}{\partial \phi }$$

Here, λ denotes the Lagrange multiplier, f refers to the free-energy density function, and σ is the surface tension coefficient.

### 2.4. Calculation Method of Slip Length

The slip length was calculated according to the approach described in Ref. [29]. Under the Navier slip boundary condition, the tangential fluid velocity $u_{x}$ at the wall is proportional to the shear rate $du_{x}/dz$; that is,

(9) $${u_{x}|}_{\left(z=0\right)}={b_{eff}(du_{x}/dz)|}_{\left(z=0\right)}$$

The effective slip length, denoted as *b*_eff_, was obtained through least-squares fitting of the cross-sectionally averaged velocity profile. It is defined as the distance from the solid wall to the point where the linearly extrapolated fluid velocity profile reaches zero.

### 2.5. Grid Convergence Test

**Figure 2:**
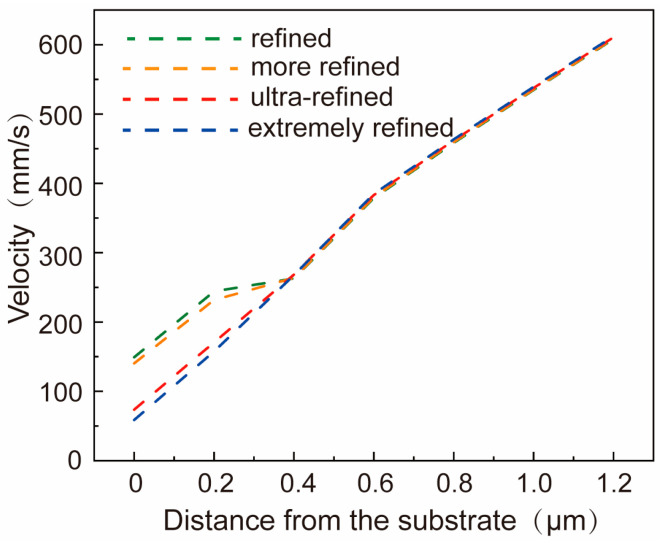
Grid independence validation.

An extra-fine mesh was employed to discretize the computational domain. Structured triangular and quadrilateral meshes were used throughout the domain, while unstructured local mesh refinement was applied in the solid–liquid and gas–liquid interfacial regions to improve interface resolution. Grid independence verification was performed using the double-layer microstructure model (Figure 1h) as a representative case. Four mesh levels were compared; as shown in Figure 2, the results demonstrate that under the extra-fine mesh configuration (the numbers of triangular mesh elements, quadrilateral mesh elements, edge elements and vertex elements are 9457, 866, 695 and 71, respectively), variations in the velocity distribution become negligible, indicating that the numerical outcomes are essentially insensitive to further increases in grid resolution.

## 3. Results and Discussion

### 3.1. Effect of Groove Number on the Continuity of Gas Film Formation and Slip Length

**Figure 3:**
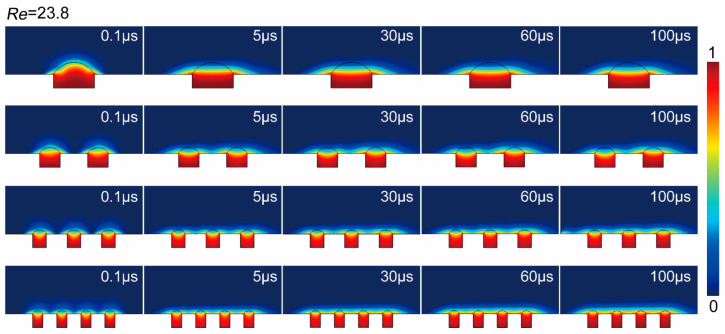
Gas film spreading behavior under different groove numbers (*Re* = 23.8). The extracted height of the microchannel is 7 μm, and the color scale represents the gas volume fraction. The wall wettability is characterized by a contact angle of 160°.

Under a constant gas–liquid ratio (*W*_G_/*W*_S_, gas–liquid contact surface area/solid–liquid contact surface area), the influence of groove number on the morphological evolution of bubbles trapped within rectangular grooves and the subsequent spreading behavior of the gas film was systematically investigated through numerical simulation. As shown in Figure 3, the dynamic process of trapped bubbles evolving into a continuous gas film is illustrated for microchannel bottom walls containing one, two, three, and four grooves, respectively. At 0 μs, each groove microstructure initially captures a bubble with a protrusion angle of 60°. Due to the small microchannel scale (*H* = 12 μm) and relatively low Reynolds number (*Re* = 23.8), thermocapillary forces dominate over the shear forces induced by forced flow. Consequently, the three-phase contact line of the bubbles spreads symmetrically toward both sides over time. At 5 μs, adjacent bubbles in the multi-groove configurations merge and connect to form a gas film. As the number of grooves increases, the resulting gas film becomes progressively more uniform and continuous.

**Figure 4:**
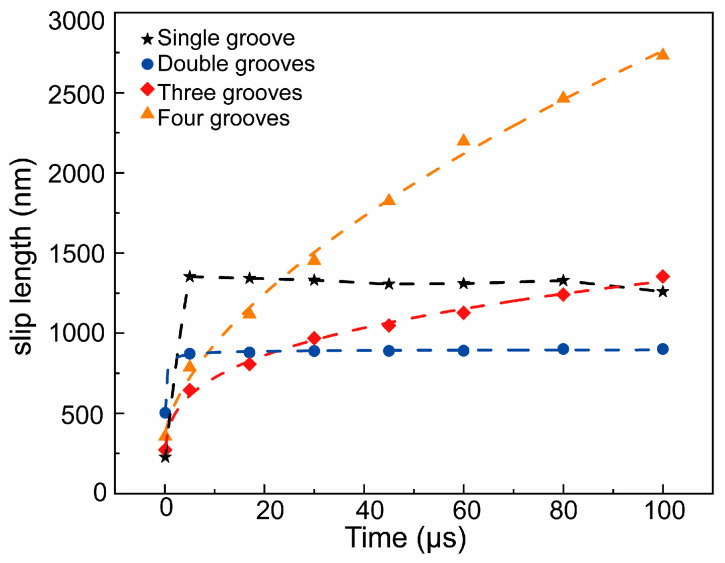
Variation in slip length during the trapped-bubble–gas film evolution process under different rectangular groove numbers.

Figure 4 illustrates the variation in slip length calculated during gas film evolution for rectangular groove configurations with groove numbers of one, two, three, and four. It can be clearly observed that the number of grooves has a pronounced effect on slip length. For the single-groove configuration, the slip length exhibits relatively minor variation throughout the entire time range. It reaches a peak value of approximately 1352 nm at 5 μs, then remains nearly constant until 80 μs, with a final value of 1230 nm at 100 μs. For the configuration of two grooves, the variation trend of slip length is generally similar to that of the single-groove case. However, due to the non-uniform gas film formed by bubble spreading between the two grooves, the slip length is significantly lower than that of the single-groove model, reaching approximately 901 nm at 100 μs. When the groove number further increases to three and four, the continuity of the gas film between adjacent grooves is gradually improved with time, leading to a correspondingly more pronounced enhancement in slip length. Under the three-groove condition, the slip length increases rapidly at 30 μs to approximately 888 nm and further rises to about 1353 nm at 100 μs.

**Figure 5:**
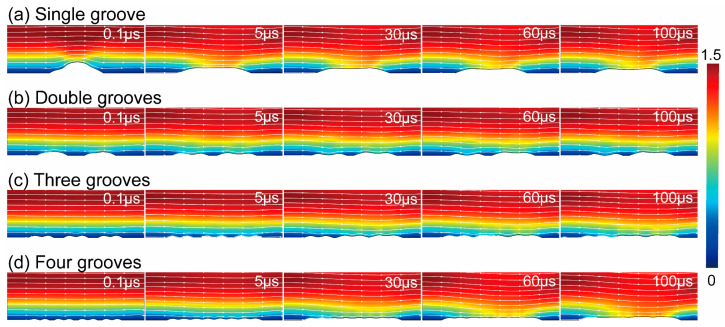
Velocity field distribution of fluid within the microchannel under different groove number conditions (*Re* = 23.8). The extracted channel height is 7 μm, and the color scale represents the velocity magnitude, with units of m/s.

For the four-groove configuration, the slip length exhibits a trend similar to that of the three-groove case, but with a much more substantial growth rate. During the initial stage (0–5 μs), the slip length rapidly increases to approximately 786 nm; it subsequently reaches about 1452 nm at 30 μs and further surges dramatically to nearly 2732 nm at 100 μs. These results demonstrate that more grooves facilitate gas aggregation and interconnection among trapped bubbles. Increasing groove number promotes the formation of a more stable and continuous gas film. This intact gas barrier isolates liquid from the solid boundary, lowers the velocity gradient near the wall and alleviates viscous dissipation, thereby significantly reducing fluid frictional resistance at the wall. The variation in slip length shown in Figure 4 is further corroborated by the velocity field evolution of the fluid during the bubble-to-gas film transition process presented in Figure 5. Overall, except for the slight superiority of the single-groove model over the two-groove model, slip length generally exhibits a progressively increasing trend with increasing groove number over time.

Our simulation results were further validated against the classical experimental data on drag reduction in superhydrophobic microchannels reported by Ou et al. [36]. Their experiments investigated the laminar flow behavior of deionized water in superhydrophobic grooved microchannels over a Reynolds number range of 10–300, which is in good agreement with the flow regime (*Re* = 23.8–238) and working fluid conditions considered in the present study. The present simulations reveal that the formation of a continuous gas film significantly increases the gas–liquid fraction, thereby enhancing the boundary slip length. This trend agrees well with the experimental observations reported in Ref. [36], where the slip length was found to increase monotonically with increasing gas–liquid ratio. Specifically, the slip length increased from approximately 5 μm to 21 μm as the gas–liquid ratio rose from 0.55 to 1. In the continuous-gas film configuration investigated in this study, a slip length of approximately 2.7 μm was obtained at a gas–liquid ratio of about 0.7. Although the absolute values differ owing to variations in surface geometry and flow conditions, the consistent dependence of slip length on the gas–liquid ratio demonstrates that the present simulations successfully capture the essential physical mechanisms governing slip enhancement in superhydrophobic microchannels. These results further confirm the reliability of the simulation results and validate the accuracy and applicability of the established numerical model for predicting the slip characteristics and drag reduction performance of superhydrophobic grooved microchannels.

### 3.2. Effect of Groove Number on the Stability of Gas Film Formation and Corresponding Variation in Slip Length

**Figure 6:**
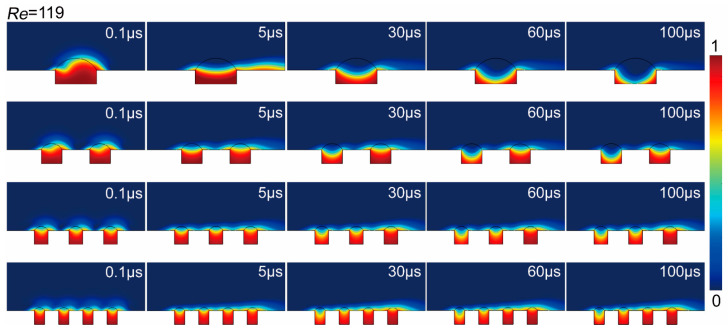
Gas film spreading behavior under different groove numbers (*Re* = 119). The extracted channel height is 7 μm with a wall contact angle of 160°.

**Figure 7:**
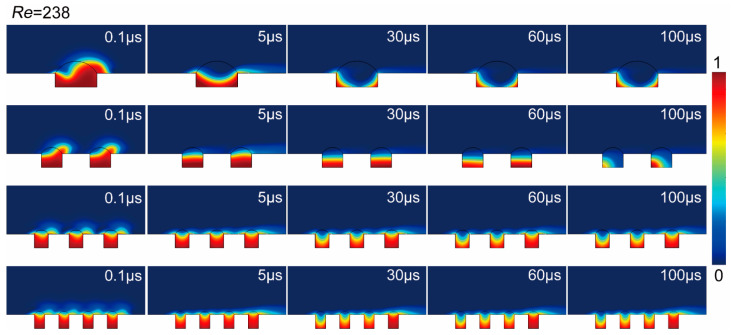
Gas film spreading behavior under different groove numbers (*Re* = 238). The extracted channel height is 7 μm with a wall contact angle of 160°.

To further investigate the influence of groove number on gas film stability, Figure 6 and Figure 7 present the bubble-to-gas film evolution processes at two different Reynolds numbers of 119 and 238, respectively. By comparison with the case of *Re* = 23.8 shown in Figure 3, it can be observed that as the Reynolds number increases to 119, the fluid shear force is significantly enhanced and becomes the dominant factor governing the gas–liquid interface dynamics. As a result, the continuity of the gas film is markedly disrupted. At 100 μs, the gas volume retained within a single groove is less than half of that observed at *Re* = 23.8. This phenomenon becomes even more severe when the Reynolds number further increases to 238. For the two-groove configuration, gas film continuity is somewhat improved at *Re* = 119; however, at *Re* = 238, only a limited portion of gas remains trapped within the rectangular microstructures at 100 μs. In contrast, when the groove number increases to three and four, although the continuity of the gas film is still partially disrupted with increasing Reynolds number, the resistance of the gas film against forced-flow shear is significantly strengthened. Even under the high-Reynolds number condition of *Re* = 238, the gas film formed in the four-groove model remains relatively stable at 100 μs and preserves a certain degree of continuity. These results indicate that increasing the number of grooves can effectively mitigate the destructive effect of local shear forces on the gas film morphology, thereby enabling the gas film to maintain greater stability under conditions of elevated shear stress.

**Figure 8:**
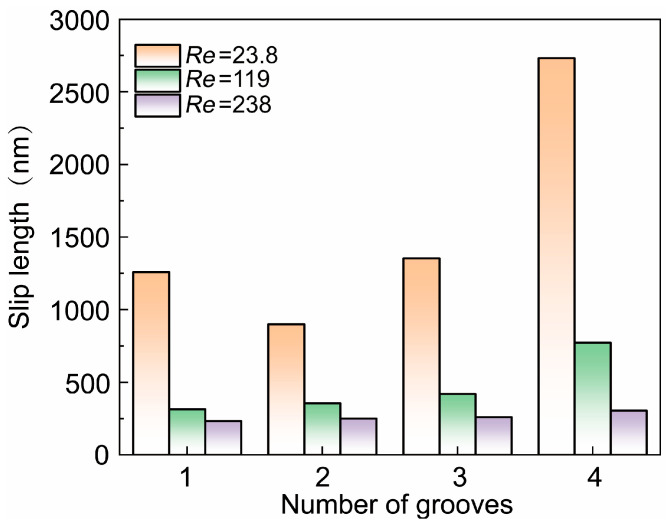
Bar chart of slip length for models with different groove numbers at 100 μs under varying Reynolds number conditions.

Figure 8 presents the bar chart of slip length for different groove numbers at Reynolds numbers of 23.8, 119, and 238. Consistent with the gas film spreading behavior observed under varying Reynolds numbers, the slip length of all models decreases with increasing Reynolds number, primarily due to the intensified shear force, which disrupts the continuity of the gas film. For models with different groove numbers at *Re* = 23.8, the slip length initially decreases as the groove number increases from a single groove to two grooves, followed by an increasing trend as the groove number further rises from two to four grooves. When the Reynolds number increases to 119 and 238, the enhanced fluid shear significantly weakens gas film continuity, resulting in an overall substantial reduction in slip length. Although slip length still increases approximately linearly with groove number under these conditions, the growth rate at *Re* = 238 decreases markedly due to stronger shear-induced gas film disruption.

**Figure 9:**
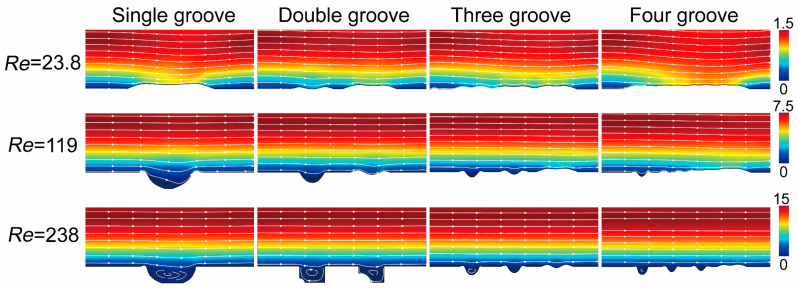
Velocity field distribution of fluid within the microchannel at 100 μs under different groove numbers and Reynolds number conditions. The extracted channel height is 7 μm, and the color scale represents the velocity magnitude, with units of m/s.

Figure 9 illustrates the fluid velocity field distributions within the microchannel at 100 μs for models with different groove numbers under Reynolds numbers of 23.8, 119, and 238. At *Re* = 23.8, the model with four grooves exhibits a larger high-velocity region compared to models with fewer grooves. As the Reynolds number increases, gas film continuity is progressively disrupted, and vortex structures begin to form within the grooves. Particularly at *Re* = 238, maintaining a stable gas–liquid interface becomes challenging for the single-groove and double-groove configurations, where the trapped gas is largely displaced by water. Owing to the reduced number of grooves, these two configurations generate significantly intensified vortices inside the groove cavities. In comparison, the three-groove and four-groove models can still effectively confine gas within the grooves, with no obvious vortex structures observed. Figure 9 visually demonstrates the influence of groove number on bubble continuity and wall–fluid boundary conditions under different Reynolds number conditions. These results further indicate that multi-groove structures can effectively enhance resistance to shear-induced gas film disruption at high Reynolds numbers, thereby sustaining superior drag reduction performance.

### 3.3. Effects of Groove Geometry and Double-Layer Microstructure on Gas Film Formation and Slip Length

**Figure 10:**
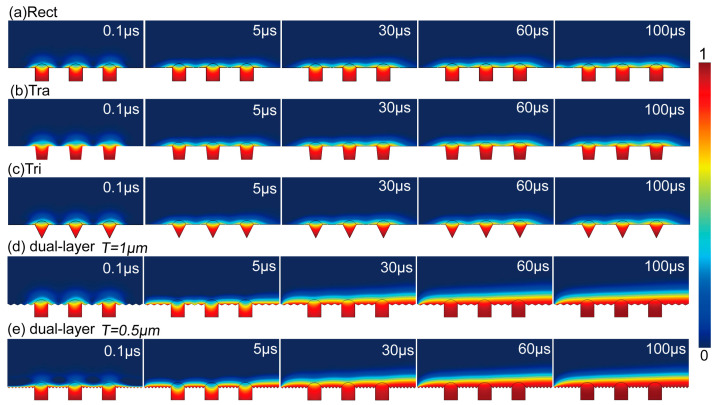
The evolution process of a gas film on channel walls with three different microstructures. (**a**) Rectangular, (**b**) trapezoidal, (**c**) triangular, (**d**) dual-layer microstructure, T = 1 μm, (**e**) dual-layer microstructure, T = 0.5 μm. The height of the microchannel interception is 7 μm and *θ*_wall_ = 160°.

Building upon the previous results, it is patently clear that a stable gas film can effectively enhance the slip length. In this section, an in-depth exploration is carried out regarding the impact of groove shape on gas film formation. As shown in Figure 10, the microstructures of the lower channel wall are, respectively, rectangular (Figure 10a), trapezoidal (Figure 10b), and triangular (Figure 10c). The Reynolds number of the fluid is set at 23.8, and the wetting angle of the wall surface is 160°. The dynamic evolution process occurs, whereby bubbles, held within grooves of different configurations, gradually evolve into gas films. It can be noted that at 5 µs, the gas film on rectangular and trapezoidal microstructures has spread completely, whereas the length of the gas film on triangular microstructures is relatively short. At 100 µs, it is evident that the spreading length of the gas film on the rectangular groove is the longest, followed by that on the trapezoidal groove and then the triangular groove. Figure 10d,e illustrate the gas film spreading evolution on the bottom wall of double-layer microstructured channels with periods of 1 μm and 0.5 μm, respectively. Compared with single-layer microstructures, the gas film on double-layer structures exhibits a noticeably longer spreading length. Moreover, the 0.5 μm microstructure model forms a continuous gas film as early as 0.1 μs, and the gas film thickness gradually increases over time once a continuous film is established. This reveals that double-layer microstructures increase wall roughness, enhance gas-trapping capacity, and improve gas film stability.

**Figure 11:**
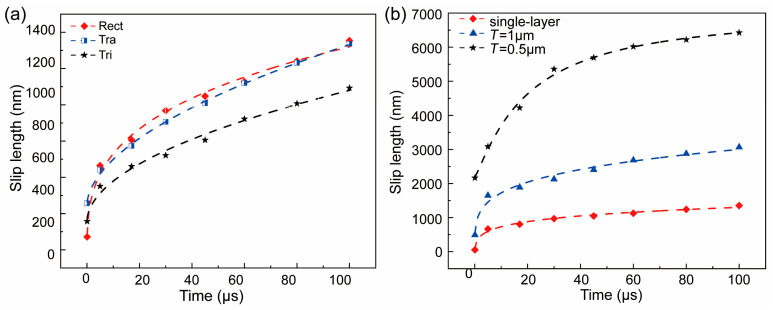
The variation curves of slip length with the evolution of the gas film on channel walls with different microstructures. (**a**) Single-layer microstructure; (**b**) Comparison between single-layer and double-layer configurations.

Figure 11 depicts the variation in the slip length in relation to the evolution of the gas film within the groove-shaped models presented in Figure 10. The variation trend in slip length is basically consistent with the spreading behavior of the gas film. Specifically, the slip length increases as the spreading length of the gas film grows. For the rectangular-groove structure surface, the slip length reaches its maximum value of 1353 nm at 100 μs (Figure 11a). By comparison, the slip length on the trapezoidal-groove channel wall is approximately 1336 nm, slightly lower than that of the rectangular groove structure. The triangular-groove surface possesses the minimum slip length, which is measured to be 1092 nm at 100 μs.

**Figure 12:**
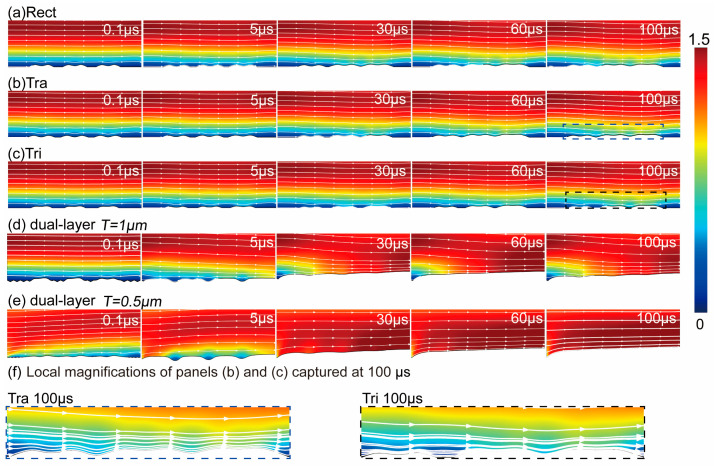
The water flow field during the evolution of gas film on channel walls with different microstructures. The height of the microchannel interception is 7 μm.

Figure 11b illustrates the variation in slip length during gas film spreading on double-layer microstructures. The slip length of double-layer microstructures is remarkably higher than that of single-layer microstructures. In addition, double-layer microstructures with a period of 0.5 μm exhibit a larger slip length than those with a period of 1 μm. At 100 μs, the slip length obtained from the model of double-layer microstructure with a period of 0.5 μm reaches 6424 nm, more than twice that of the counterpart with a period of 1 μm (3066 nm) and approximately 4.75 times higher than that of the single-layer microstructure model (1353 nm). The bilayer structure achieves multiscale superposition of base and tiny protrusions, constructing abundant hierarchical concave spaces. This distinctive surface morphology can effectively capture steady gas layers and firmly anchor the three-phase contact line, restraining interfacial distortion and near-wall flow disturbance. Benefiting from the optimized gas film continuity and thickness, slip length is greatly elevated, which substantially cuts down flow frictional resistance and maintains a stable drag reduction effect. It should be noted that the slip length measured for the gas film is comparable to the channel characteristic size (the channel height *h* = 12 μm). Neglecting slip behavior will cause up to 81% deviation [36] in flow resistance. Figure 12 further provides supportive flow field evidence by demonstrating the water flow field distribution during gas film formation on channel walls with different microstructures.

## 4. Conclusions

This study employs numerical simulations to systematically investigate the effects of groove number, groove morphology, and double-layer microstructure on the bubble-to-gas film evolution process and fluid slip length in superhydrophobic microchannels. The results reveal that increasing the groove number significantly enhances the continuity and stability of gas film spreading. The groove geometry also plays a critical role in modulating the spreading behavior. Among the configurations examined, rectangular grooves yield the largest gas film spreading length and the best drag reduction performance, whereas triangular grooves exhibit a relatively limited gas film coverage area. Furthermore, double-layer microstructures effectively increase gas film thickness and improve its stability. A double-layer microstructure with a smaller period further accelerates gas film spreading and enhances film thickness, leading to a substantial improvement in wall slip length and drag reduction performance. Specifically, the slip length achieved by the double-layer microstructure with a period of 0.5 μm reaches 6424 nm, which is more than twice that of the 1 μm-period counterpart (3066 nm) and approximately 4.75 times higher than that of the single-layer microstructure (1353 nm). This work elucidates the intrinsic relationship between geometric parameters of microstructures and gas film-induced drag reduction performance. It provides theoretical guidance for the structural design of microfluidic chip channels and the drag reduction performance improvement of MEMS-based microfluidic devices, thereby contributing to improved operational performance and efficiency of microfluidic systems.

## Figures and Tables

**Table 1:** Core parameters of the microchannel numerical model.

Reynolds Number	Inlet Velocity	Channel Dimensions	Operating Temperature
23.8–238	1.0–10.0 m/s	Total length 20 μm	293.15 K (20 °C)
		Total height 12 μm	

## Data Availability

The original contributions presented in this study are included in the article. Further inquiries can be directed to the corresponding author.
